# Overcoming Diagnostic Uncertainty: A Case of Temporal Lobe Epilepsy With Normal MRI and EEG

**DOI:** 10.7759/cureus.71125

**Published:** 2024-10-09

**Authors:** Muhammad Taimour Khan, Nazar Muhammad, Muhammad Rahab Khan, Monika Bai, Partab Rai

**Affiliations:** 1 Internal Medicine, Combined Military Hospital (CMH) Lahore Medical College and Institute of Dentistry, Lahore, PAK; 2 Psychiatry, Cornerstone Family Healthcare, New York, USA; 3 Internal Medicine, IK Akhunbaev Kyrgyz State Medical Academy, Bishkek, KGZ; 4 Obstetrics and Gynaecology, Shaikh Zaid Women Hospital, Larkana, PAK; 5 Medicine, NYC Health + Hospitals, New York, USA

**Keywords:** auditory hallucination, diagnostic testing, electroencephalography (eeg), epilepsy, temporal lobe and neuro-psychiatric manifestations

## Abstract

Temporal lobe epilepsy (TLE) can present in a variety of ways and is challenging to diagnose. While MRIs are the gold standard for detecting structural lesions and EEGs can identify epileptiform discharges, relying solely on these diagnostic methods can result in underdiagnosis or misdiagnosis of the condition. Seizures with motor components may manifest as unusual behavior; anxiety, psychosis, and other psychiatric conditions can occur in conjunction with or independently of seizure activity. In cases where diagnostic testing yields negative results but the history strongly suggests seizure-like activity, practitioners should not hesitate to initiate antiepileptic medications.

Here, we present the case of a patient who exhibited behavioral problems, including shouting religious slogans and experiencing recurrent loss of consciousness, either occurring together or separately, while lacking awareness. The patient had a history of type 2 diabetes and no relevant family history. An extensive multidisciplinary evaluation was performed, including EEG and MRI, all of which returned normal results. Given the high clinical suspicion of TLE, the patient was started on the antiepileptic medication divalproex sodium. After six months of treatment, there was a significant improvement in all symptoms. This case highlights the importance of considering TLE in patients with suggestive clinical features even when initial diagnostic tests are normal and underscores the potential efficacy of early antiepileptic treatment in managing symptoms effectively.

## Introduction

Temporal lobe epilepsy (TLE) is a focal form of epilepsy that predominantly affects one side of the temporal lobe and is the most common type of focal epilepsy [1,2]. This condition can be classified as either idiopathic or symptomatic epilepsy [3]. Symptomatic TLE can be further divided into three categories: (1) mesial temporal lobe epilepsy (MTLE), which involves the medial side of the temporal lobe, including the hippocampus; (2) lesional TLE, which involves areas connected to the medial side and produces seizures similar to those seen in MTLE; and (3) cryptogenic TLE, where the underlying cause remains undetermined [3]. Recently, classification has shifted focus to the anatomical origin of the epileptic electrical signal within the temporal lobe, distinguishing between MTLE and neocortical (or lateral) TLE, which affects the lateral aspect of the temporal lobe [1].

The International League Against Epilepsy's classification of seizures aids in identifying seizure types and tailoring management. Focal seizures are categorized as focal onset aware seizures, in which the individual retains awareness or focal onset impaired awareness seizures, where awareness is compromised or confusion occurs. These classifications were previously known as simple partial seizures and complex partial seizures, respectively [2]. Additionally, focal onset seizure symptoms can be classified as motor or non-motor [2].

Temporal lobe epilepsy represents the most common form of focal epilepsy, and focal epilepsy accounts for approximately 60% of all adult epilepsy cases. However, the true epidemiology of TLE is often underestimated due to diagnostic challenges, including the necessity for advanced neuroimaging, positive EEG results, and accurate clinical semiology [4]. The term 'partial epilepsy' has been replaced with 'focal epilepsy' because 'focal' more accurately describes the origin of seizures from a specific area or group of cells in one hemisphere of the brain [2]. Furthermore, the underrepresentation of incidental mesial temporal lobe sclerosis in healthy individuals may also affect true epidemiological estimates [4].

Diagnosis of TLE typically relies on clinical evaluation, supplemented by MRI to visualize medially affected areas, such as hippocampal sclerosis, or EEG to detect abnormal electrical activity in the temporal lobe's cortical regions, often manifesting as spikes [1]. However, both EEG and MRI have limitations. For instance, MRI findings can be normal in up to 30% of patients with TLE [5].

This case involves a patient with a high clinical suspicion of TLE despite inconclusive test results and negative findings following a multidisciplinary assessment. This underscores the diagnostic challenges associated with TLE and highlights the need to consider it as a potential diagnosis in patients presenting with atypical symptoms. The patient's positive response to divalproex sodium and subsequent symptom improvement strengthens the diagnosis of atypical presentations of TLE and reinforces the importance of maintaining a high index of suspicion in similar cases. These findings illustrate the potential for TLE to present with complex behavioral and cognitive symptoms, offering valuable insights into managing such cases when standard diagnostic tools are insufficient.

## Case presentation

The patient self-referred to the outpatient clinic after experiencing recurrent worsening episodes of behavioral problems (including calling out religious slogans) and recurrent loss of consciousness lasting less than five minutes. At times, the behavioral problems and loss of consciousness occurred together, while at other times, they were separate events, occurring days or even weeks apart from each other. His past medical history includes chronic, intermittent headaches for more than 15 years and diabetes mellitus type II for five years. His medications consist of metformin (1000 mg twice daily) and as-needed paracetamol for the headaches. He has no previous psychiatric history of mental illness and has never used or been prescribed any psychotropic medication. He also has no personal or family history of tonic-clonic seizures or cardiac issues and reported no instances of febrile seizures as a child. The patient is a married 55-year-old male of average height and weight, of Indian origin. He works as a successful businessman and has three children.

Notably, the patient had 10 previous hospitalizations for worsening behavioral problems and episodes of loss of consciousness. The behavioral problems were characterized by outbursts of loudly calling out the same religious slogans repeatedly, lasting for two to three minutes, with no awareness of his surroundings. During these episodes, when his family intervened, he would be belligerent and aggressive. At other times, during periods of stress, he experienced recurrent loss of consciousness lasting about five minutes, without postictal confusion.

At intake, he was taking metformin 1000 mg twice daily and as-needed paracetamol for intermittent headaches. A review of the hospital admission records showed that the patient lost awareness of his surroundings during episodes of behavioral outbursts. He repeatedly shouted the religious slogan "Ya Ali" several times a week. His family noted that he experienced a brief loss of consciousness lasting about five minutes without any confusion after encountering minor psychosocial stressors, on average once or twice a month. When questioned, the patient lacked awareness of both the behavioral problems and the loss of consciousness.

A review of psychiatric symptoms for potential psychiatric illness was negative. The patient reported no biological or somatic symptoms of depression and reported no changes in appetite or sleep, with minimal psychosocial stressors. He also denied mood swings, symptoms of hypomania or mania, and anxiety symptoms. There were no observed or reported symptoms of psychotic disorders, such as hallucinations, delusions, or disorganized thinking, during the intake evaluation. He also denied any history of significant physical trauma, head injury, or other accidents that could contribute to his current mental health status.

Considering the patient's history of diabetes mellitus and the use of metformin, a concern for recurrent hypoglycemia was considered. A rapid blood glucose test returned normal results, and his metformin levels were within the therapeutic range. Routine lab tests, including CBC, comprehensive metabolic panel (CMP), thyroid-stimulating hormone (TSH), and hemoglobin A1c (HbA1c), also returned normal results. A rapid drug test for illicit drug use was conducted to explain his aggressive behavior; however, the patient denied using any illicit drugs, and the test results were negative. As part of the differential diagnosis, we considered syncopal episodes due to cardiac irregularities as a potential cause for the loss of consciousness. The patient was referred to cardiology, where he underwent an EKG and echocardiogram, both of which returned negative results. Given the persistent headache and unusual behavior, we suspected organic lesions in the brain or the possibility of tumors. Neurology was consulted, and both an MRI and an EEG were performed. Both results were normal, and based on these findings, neurology did not recommend starting the patient on any medications.

Considering that all standard laboratory tests were normal, including the brain MRI, EEG, and cardiology evaluations ruling out the possibility of syncopal episodes, no diagnostic evidence pointed to an acute or chronic cause. We suspected that the combination of unusual behavior, including shouting religious slogans, aggressive and belligerent behavior, and lack of awareness of surroundings with and without loss of consciousness, might indicate TLE. Although the MRI and EEG results were negative, the patient was diagnosed with TLE and started on divalproex sodium delayed-release 500 mg twice daily. After more than six months of medication compliance, the patient's behavioral symptoms, headaches, and loss of consciousness improved dramatically, with rare occurrences of all symptoms. Table 1 summarizes our findings.

**Table 1:** Overview of patient findings CMP: Comprehensive metabolic panel, TSH: Thyroid-stimulating hormone, HbA1c: Hemoglobin A1c, TLE: Temporal lobe epilepsy

Category	Details
Patient Info	Age/gender: 55/male; Ethnicity: Indian; Occupation: Businessman; Marital status: Married with three children
Symptoms	Behavioral problems (calling out religious slogans), loss of consciousness (<5 minutes), chronic headaches
Medical history	Headaches (>15 years), type II diabetes (five years)
Medications	Metformin 1000 mg (twice a day), paracetamol (as needed)
Psychiatric history	No psychiatric illness or psychotropic medication use, no personal/family history of seizures or cardiac issues
Hospitalizations	10 episodes for worsening behavioral problems and loss of consciousness
Behavioral episodes	Religious outbursts lasting for two to three minutes, aggressive behavior during episodes, stress-induced unconsciousness (~5 minutes)
Diagnostics	Labs: Normal glucose, CBC, CMP, TSH, HbA1c, and drug test; Cardiac: Normal EKG and echo; Neuro: Normal MR and EEG
Diagnosis	TLE
Treatment	Divalproex sodium 500 mg twice a day. Significant symptom improvement was observed after six months.

## Discussion

Despite no previous psychiatric history and normal results from blood tests, brain MRI, EEG, and cardiac evaluations in our 55-year-old patient, his symptoms persisted. Notably, he had a pattern of aggressive outbursts and loss of consciousness, occasionally linked to stress. The differential diagnosis considered hypoglycemia, cardiac issues, and organic brain lesions, but these were ruled out. Based on the clinical presentation, including the behavioral outbursts and lack of awareness, TLE with focal onset impaired awareness seizures was suspected, although initial tests were normal. The patient was prescribed divalproex sodium, which led to significant improvement in his symptoms over six months, with a notable reduction in both behavioral issues and episodes of loss of consciousness.

Temporal lobe epilepsy can originate from either the medial or outer part of the temporal lobe. An MRI showing hippocampal sclerosis in the medial part or an EEG showing an epileptiform discharge in the outer part are diagnostic [1,6]. In the EEG findings of MTLE due to hippocampal sclerosis, interictal discharges are seen in the anterior/mid-temporal spikes, being ipsilateral or predominant over the affected temporal lobe, while during the seizures, the EEG typically reveals a rhythmic 5-7 Hz discharge [6]. However, it should be noted that patients with TLE are termed 'oligospikers' when they show little to no interictal epileptiform discharges (IEDs) on their respective EEGs [5].

The EEG is used by placing electrodes outside the body to detect electrical signals in the brain. If the signal is epileptic, the EEG will record these findings. In TLE, it primarily detects signals from the outer part of the temporal lobe and can only detect abnormal nerve activity when it involves a sufficiently large area. However, signals detected by EEG can often be misleading, as they may appear to originate from one brain region when they come from another due to the spread of electrical activity along specific pathways or through the brain's fluid. Additionally, the origins of many normal and abnormal EEG signals remain unclear. Moreover, standard scalp electrode placement does not cover all brain areas, particularly deeper regions, leaving some critical zones unmonitored. Lastly, because EEG recordings are typically brief, they may miss abnormal brain activity that does not occur during the recording session, which is why a person with epilepsy might have a normal EEG result even if they have the condition. Hence, these challenges and limitations are encountered when using an EEG [6].

In MTLE, hippocampal sclerosis is a condition where the hippocampus on one or both sides has shrunken, a common finding on MRI [1]. However, MRI is negative in 30% of patients with TLE, making it difficult to identify an epileptogenic zone. Some still regard this as MTLE, while others consider it a separate entity [5].

Oligospikers with MRI-negative TLE tend to have less severe epilepsy compared to patients with frequent IEDs, but the surgical outcome remains the same between oligospikers and frequent spikers. However, those with unilateral IEDs will show better outcomes as well as patients with IEDs in the posterior part of the temporal lobe compared to those with anterior or bilateral IEDs. This highlights the need to identify and treat these patients, even when MRI or EEG results are normal [5].

In cases where MRI and EEG are normal, further diagnostic evaluations can be performed to investigate structural concerns that may be triggering epilepsy. The increased use of high-field 3 Tesla (3T) MRI scans has led to the reclassification of many cases that were previously labeled as MRI-negative into the lesion-positive category. Moreover, small temporal pole encephaloceles are now recognized as a previously overlooked cause of MRI-negative TLE, and emerging cases of TLE associated with enlargement of the ipsilateral amygdala have recently been identified as a potential subtype of MRI-negative TLE. An intracranial EEG (ICEEG) can determine whether the hippocampus is involved in seizure generation, and can guide a surgery that spares the hippocampus, thereby reducing postoperative risks. Moreover, ICEEG is useful in ruling out bitemporal, pseudotemporal, or temporal-plus epilepsies, which typically have worse surgical outcomes than anterior TLE [5]. Functional MRI (fMRI) can also be used; however, it shows different patterns of brain connectivity in MRI-negative TLE compared to TLE with hippocampal sclerosis. In MRI-negative TLE, there is a decrease in connectivity of the ipsilateral temporal neocortex, while the mesial temporal structures are relatively spared [5]. 

Proton magnetic resonance spectroscopy (MRS) detects metabolic alterations in patients with TLE, but PET scans or single-photon emission computed tomography (SPECT) are more commonly preferred, especially in the presurgical evaluation of MRI-negative TLE. The PET scans, using 2-(18F)fluoro-2-deoxy-d-glucose positron emission tomography (FDG-PET) and other ligands, can identify metabolic disturbances related to epilepsy when MRI is negative. On the other hand, 99m Tc-hexamethyl propylene amine oxime (99mTc-HMPAO) SPECT and 99m Tc-ethyl cysteinate dimer (99mTc-ECD) provide an indirect assessment of cerebral blood flow (CBF) changes, localizing seizures during interictal and ictal states, especially preoperatively. Therefore, proton MRS, PET, and SPECT are useful in detecting metabolic and CBF changes associated with seizure activity in MRI-negative TLE [5].

People with epilepsy are often observed to have strong spiritual feelings or experiences related to religion, with some even developing a deep connection with religion after a seizure. One possible explanation is that during an epileptic episode, an intense religious feeling experienced during an aura, followed by confusion afterward, may lead some to become fixated on religion and God [7]. People with TLE have been observed to exhibit Geschwind syndrome, in which profound spiritual experiences are a common trait. A study suggested that neurological, psychiatric, and cultural factors might influence these spiritual feelings instead [7]. Another study found no conclusive link between epilepsy and religion, noting that much of the early literature suggesting such connections may have overstated the evidence [8,9].

Various psychiatric conditions, such as anxiety, psychosis, and aggression, are seen in epilepsy. These conditions are categorized as peri-ictal, where psychiatric events occur before or after an ictal period. Ictal refers to the psychiatric clinical expression of a seizure, and interictal is when it occurs independently of seizures. Psychiatric symptoms are thus classified according to their temporal relationship with seizures [10]. Recognizing psychiatric conditions as symptoms of epilepsy is crucial, as 50% to 60% of people with epilepsy have psychiatric problems. One method to confirm this includes using video-EEG monitoring, which documents electrical seizure activity during a psychiatric episode, thereby confirming an epileptic episode [11].

Infants with TLE commonly exhibit motor manifestations, which decrease as they reach adulthood. However, automatisms are the most common manifestation across all age groups and tend to increase with age [12]. Common motor symptoms include clonic, atonic, tonic, and myoclonic movements, as well as repeated movements known as automatisms, which typically involve actions like clapping or hand rubbing. Non-motor symptoms involve changes in sensations, emotions, cognition, autonomic functions, and behavioral arrest [2].

## Conclusions

This case underscores the complexity of diagnosing TLE, particularly in instances where conventional imaging and EEG tests are inconclusive. Despite normal results from EEG and MRI, the patient’s clinical presentation, characterized by an unusual behavior of shouting religious slogans, loss of consciousness, and lack of awareness, suggested TLE; suspected epileptic seizures should always undergo a thorough assessment. Initiating treatment with divalproex sodium led to a marked improvement in symptoms, affirming the value of clinical judgment in the face of negative diagnostic tests. This case emphasizes the need for a high index of suspicion for TLE in patients with suggestive clinical features and supports the efficacy of early antiepileptic intervention in achieving symptom relief.
